# Supplementary material for: Evaluation of Blood Levels of Omentin-1 and Orexin-A in Adults with Obstructive Sleep Apnea: A Systematic Review and Meta-Analysis
Source: Life (Basel). 2023 Jan 16;13(1):245. doi: 10.3390/life13010245 (PMC9865616; doi:10.3390/life13010245)
Supplement: Supplementary file 1 [file life-13-00245-s001.zip › life-2091899-supplementary.pdf]

**Table S1**: The Joanna Briggs Institute (JBI) Critical Appraisal Checklist for case-control studies

| **I. The Joanna Briggs Institute (JBI) Critical Appraisal Checklist for case-control study (last amended in 2017)**<br>**Website:** https://joannabriggs.org/critical_appraisal_tools<br>https://wiki.joannabriggs.org/display/MANUAL/Appendix+7.2+Critical+appraisal+checklist+for+case-control+studies | | | | |
|---|---|---|---|---|
| Major Components | Response options | | | |
| 1. Were the groups comparable other than the presence of disease in cases or the absence of disease in controls? | Yes | No | Unclear | Not applicable |
| 2. Were cases and controls matched appropriately? | Yes | No | Unclear | Not applicable |
| 3. Were the same criteria used for identification of cases and controls? | Yes | No | Unclear | Not applicable |
| 4. Was exposure measured in a standard, valid and reliable way? | Yes | No | Unclear | Not applicable |
| 5. Was exposure measured in the same way for cases and controls? | Yes | No | Unclear | Not applicable |
| 6. Were confounding factors identified? | Yes | No | Unclear | Not applicable |
| 7. Were strategies to deal with confounding factors stated? | Yes | No | Unclear | Not applicable |
| 8. Were outcomes assessed in a standard, valid and reliable way for cases and controls? | Yes | No | Unclear | Not applicable |
| 9. Was the exposure period of interest long enough to be meaningful? | Yes | No | Unclear | Not applicable |
| 10. Was appropriate statistical analysis used? | Yes | No | Unclear | Not applicable |

| Study name | Statistics with study removed | | | | | | |
|---|---|---|---|---|---|---|---|
| | Point | Standard error | Variance | Lower limit | Upper limit | Z-Value | p-Value |
| Blçer, 2021 | -0.98 | 0.82 | 0.68 | -2.59 | 0.63 | -1.19 | 0.23 |
| Kurt, 2014 | -1.08 | 0.77 | 0.59 | -2.58 | 0.42 | -1.41 | 0.16 |
| Uygur, 2016 | -0.58 | 0.82 | 0.68 | -2.19 | 1.03 | -0.71 | 0.48 |
| Wang, 2013 | -0.36 | 0.47 | 0.22 | -1.27 | 0.56 | -0.76 | 0.45 |
| Zhang, 2018 | -0.88 | 0.81 | 0.66 | -2.46 | 0.71 | -1.08 | 0.28 |
| Zirlik, 2013 | -1.15 | 0.75 | 0.57 | -2.62 | 0.32 | -1.53 | 0.13 |
| | -0.84 | 0.69 | 0.48 | -2.20 | 0.52 | -1.21 | 0.23 |

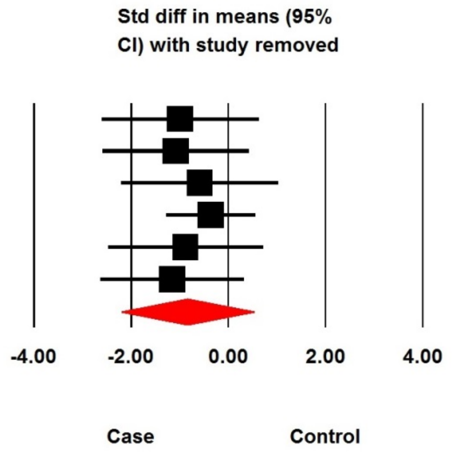


**Figure S1**: One-study-remove analysis of blood omentin-1 levels in adults with OSA compared to controls

| Study name | Cumulative statistics | | | | | | |
|---|---|---|---|---|---|---|---|
| | Point | Standard error | Variance | Lower limit | Upper limit | Z-Value | p-Value |
| Blçer, 2021 | -0.12 | 0.16 | 0.03 | -0.44 | 0.20 | -0.74 | 0.46 |
| Kurt, 2014 | 0.10 | 0.24 | 0.06 | -0.37 | 0.56 | 0.40 | 0.69 |
| Uygur, 2016 | -0.61 | 0.67 | 0.45 | -1.92 | 0.71 | -0.91 | 0.36 |
| Wang, 2013 | -1.28 | 0.90 | 0.81 | -3.04 | 0.48 | -1.42 | 0.15 |
| Zhang, 2018 | -1.15 | 0.75 | 0.57 | -2.62 | 0.32 | -1.53 | 0.13 |
| Zirlik, 2013 | -0.84 | 0.69 | 0.48 | -2.20 | 0.52 | -1.21 | 0.23 |
| | -0.84 | 0.69 | 0.48 | -2.20 | 0.52 | -1.21 | 0.23 |

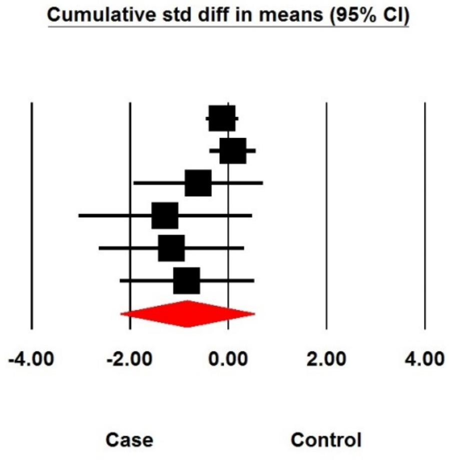


**Figure S2**: Cumulative analysis of blood omentin-1 levels in adults with OSA compared to controls

| Study name | Point | Standard error | Variance | Lower limit | Upper limit | Z-Value | p-Value |
|---|---|---|---|---|---|---|---|
| | Statistics with study removed | | | | | | |
| Zhu, 2022 | 0.08 | 0.42 | 0.18 | -0.75 | 0.91 | 0.18 | 0.85 |
| Yao, 2006 | -0.32 | 0.58 | 0.34 | -1.46 | 0.82 | -0.55 | 0.58 |
| Oztürk, 2022 | -0.40 | 0.60 | 0.36 | -1.57 | 0.77 | -0.67 | 0.51 |
| Igarashi, 2003 | -0.34 | 0.57 | 0.33 | -1.46 | 0.78 | -0.60 | 0.55 |
| Busquets, 2004 | 0.25 | 0.45 | 0.20 | -0.63 | 1.13 | 0.55 | 0.58 |
| Liao, 2005 (i) | -0.44 | 0.55 | 0.30 | -1.51 | 0.64 | -0.79 | 0.43 |
| Liao, 2005 (ii) | -0.41 | 0.56 | 0.32 | -1.51 | 0.70 | -0.73 | 0.47 |
| Strueva, 2015 | -0.12 | 0.54 | 0.29 | -1.18 | 0.94 | -0.23 | 0.82 |
| | -0.21 | 0.50 | 0.25 | -1.19 | 0.77 | -0.42 | 0.67 |

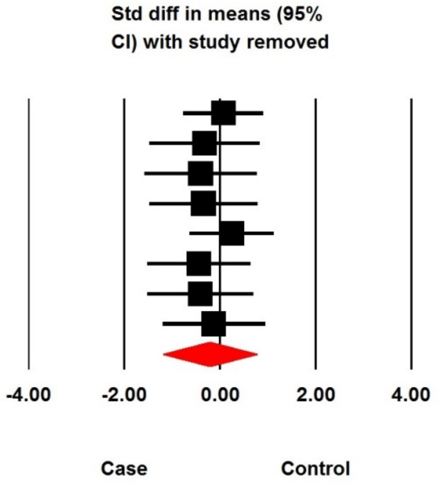


**Figure S3**: One-study-remove analysis of blood orexin-A levels in adults with OSA compared to controls

| Study name | Point | Standard error | Variance | Lower limit | Upper limit | Z-Value | p-Value |
|---|---|---|---|---|---|---|---|
| | Cumulative statistics | | | | | | |
| Zhu, 2022 | -1.98 | 0.26 | 0.07 | -2.48 | -1.47 | -7.63 | 0.00 |
| Yao, 2006 | -0.75 | 1.23 | 1.50 | -3.15 | 1.65 | -0.61 | 0.54 |
| Oztürk, 2022 | -0.17 | 0.89 | 0.80 | -1.92 | 1.58 | -0.19 | 0.85 |
| Igarashi, 2003 | 0.03 | 0.67 | 0.45 | -1.28 | 1.34 | 0.05 | 0.96 |
| Busquets, 2004 | -0.68 | 0.72 | 0.52 | -2.10 | 0.74 | -0.94 | 0.35 |
| Liao, 2005 (i) | -0.34 | 0.63 | 0.39 | -1.57 | 0.89 | -0.54 | 0.59 |
| Liao, 2005 (ii) | -0.12 | 0.54 | 0.29 | -1.18 | 0.94 | -0.23 | 0.82 |
| Strueva, 2015 | -0.21 | 0.50 | 0.25 | -1.19 | 0.77 | -0.42 | 0.67 |
| | -0.21 | 0.50 | 0.25 | -1.19 | 0.77 | -0.42 | 0.67 |

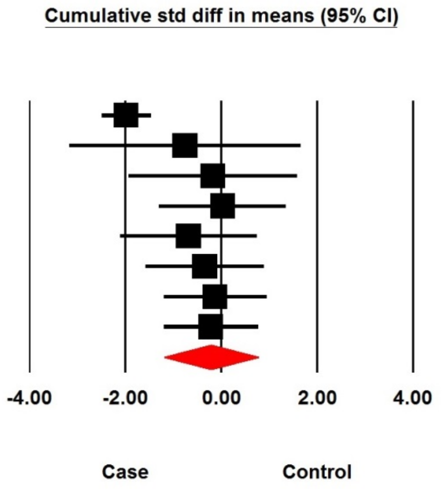


**Figure S4**: Cumulative analysis of blood orexin-A levels in adults with OSA compared to controls
